# Deletion of the Toll-Like Receptor 5 Gene Per Se Does Not Determine the Gut Microbiome Profile That Induces Metabolic Syndrome: Environment Trumps Genotype

**DOI:** 10.1371/journal.pone.0150943

**Published:** 2016-03-07

**Authors:** Wei Zhang, Riley Hartmann, Hein Min Tun, Charles O. Elson, Ehsan Khafipour, W. Timothy Garvey

**Affiliations:** 1 Department of Nutrition Sciences, School of Health Professions, the University of Alabama at Birmingham, Birmingham, Alabama, United States of America; 2 Department of Medical Microbiology and Infectious Diseases, University of Manitoba, Winnipeg, Canada; 3 Department of Animal Science, University of Manitoba, Winnipeg, Canada; 4 Division of Gastroenterology and Hepatology, School of Medicine, the University of Alabama at Birmingham, Birmingham, Alabama, United States of America; 5 Birmingham VA Medical Center, Birmingham, Alabama, United States of America; Institut d'Investigacions Biomèdiques August Pi i Sunyer, SPAIN

## Abstract

Over the past decade, emerging evidence has linked alterations in the gut microbial composition to a wide range of diseases including obesity, type 2 diabetes, and cardiovascular disease. Toll-like receptors (TLRs) are the major mediators for the interactions between gut microbiota and host innate immune system, which is involved in the localization and structuring of host gut microbiota. A previous study found that TLR5 deficient mice (TLR5KO1) had altered gut microbial composition which led to the development of metabolic syndrome including hyperlipidemia, hypertension, insulin resistance and increased adiposity. In the current study, a second TLR5-deficient mouse model was studied (TLR5KO2). TLR5 deficient mice did not manifest metabolic abnormalities related to the metabolic syndrome compared with littermate controls maintained on normal chow or after feeding a high fat diet. Analysis of the gut microbial composition of littermate TLR5KO2 and wild type mice revealed no significant difference in the overall microbiota structure between genotypes. However, the TLR5KO2 microbiota was distinctly different from that previously reported for TLR5KO1 mice with metabolic syndrome. We conclude that an altered composition of the microbiota in a given environment can result in metabolic syndrome, but it is not a consequence of TLR5 deficiency per se.

## Introduction

In humans and other mammals, the gut microbiome is essential for physiological homeostasis, and, under normal conditions, protects against pathogens and enhances food digestion and nutrient absorption. Gut microbiota are transmitted maternally, but host genetics and environmental factors also modify and shape microbiota composition. Alterations in the gut microbiome can also participate in disease, and have been associated with host metabolic abnormalities including inflammatory bowel disease [1], obesity, insulin resistance[2], diabetes[3, 4] and cardiovascular disease[5]. During the past decade, signaling pathways have been identified to show that gut microbiota negatively impact host physiology, particularly regarding disease processes involving insulin resistance or chronic inflammation.

Host innate immunity is directly involved in the localization and structuring of host gut microbiota[6]. Toll-like receptor 5 (TLR5) is an innate immunity receptor that recognizes bacterial flagellin and is highly expressed on the intestinal mucosa. A previous study reported that TLR5 deficient mice (TLR5KO1) had alterations in gut microbiota composition that resulted in metabolic syndrome including hyperlipidemia, hypertension, insulin resistance, and increased adiposity[7]. Moreover, similar metabolic changes occurred upon transfer of microbiota from TLR5KO1 to wild type mice. In the current study, a second mouse line with TLR5 gene deficiency (TLR5KO2) was studied with the objective to understand the mechanisms by which TLR5 deficiency can cause host insulin resistance. Contrary to our expectations, the metabolic profile of littermate TLR5KO2 mice and wild type control mice were the same despite multiple dietary challenges. Since environmental factors and host genetics both participate in the modulation of gut microbiota, we analyzed the gut microbial composition of TLR5KO2 mice and compared these data to those previously reported in the TLR5KO1 mice[7]. Because both lines had TLR5 deficiency but were raised in different environments, the data distinguish the interactions between gut microbiota and host innate immunity from the interactions between gut microbiota and environment. Furthermore, these results provide new insights into the role of certain microbes in the development of obesity and insulin resistance in the host.

## Methods & Materials

### Generation and management of animal models

The TLR 5 knockout mice (TLR5KO2) mouse model was a gift from Dr.Richard Flavell, Yale University, and generated as previously described[8]. All of the animals were housed in a specific pathogen-free animal facility with 12-h light/dark cycles, and received a standard laboratory chow diet unless otherwise indicated for the high fat diet experiments. Both male and female mice were studied in the experiments, and were maintained with *ad lib* access to water and either regular chow (18% protein, 46.5% carbohydrate, 4.7%fat by weight, 24% protein, 62% carbohydrate, 14% fat by calorie; Teklad 7917, Harlan Laboratories, Madison, WI.), or to a high fat diet (26.2% protein, 26.3% carbohydrate, 34.9% fat by weight, 20% protein, 20% carbohydrate, 60% fat by calorie; D12492, Research Diets Inc., New Brunswick, NJ). To reduce the possibility that gut microbial composition could be cross-contaminated between wild type and knockout mice, the metabolic studies were repeated on mice that were segregated and caged by different genotypes since the time of weaning and on mice that were caged without segregating by genotypes. Moreover, TLR5KO2 mice were crossed to B6 wild type mice and the F1 generation was intercrossed to generate littermate TLR5 knockout (TLR5 KO) and wild type (WT) mice for comparisons. All animal procedures were approved by the Institutional Animal Care and Use Committee (IACUC) of the Animal Resources Program (ARP) at the University of Alabama at Birmingham.

### Experimental design

In the previous study[7], body weight under chow diet (fat 4.5%, Lab Diets 5001) was recorded to 20 weeks of age, magnetic resonance imaging (MRI) was applied for body composition measurement at 20 weeks of age. For high fat diet feeding, four-week-old WT and TLR5KO mice were given a high-fat diet (fat 34.9%, D12492, Research Diets Inc., New Brunswick, NJ) for 8 weeks starting from 4 weeks of age. To avoid the confounding effects of co-housing on the diversity of cecal bacteria, mice from multiple litters that were housed separately were selected for gut microbiome analysis. In the current study, body weight under chow diet was recorded to 20 weeks of age, for high fat feeding, mice were given a high-fat diet (fat 34.9%, D12492, Research Diets Inc., New Brunswick, NJ) for 12 weeks starting from 8 weeks of age. Quantitative magnetic resonance imaging (QMR) was applied for body composition measurement at 20 weeks of age. To avoid the effects of co-housing on gut microbiome, multiple litters that were separately caged since weaning were selected for gut microbiome analysis.

### General PCR and Real-time PCR experiment confirmation of knockout

Genotypes were determined using the following primers to analyze tail DNA: wild type antisense: 5’-TGAACAAACACTGCCTGCGTG-3’; wild type sense: 5’-AACACCACATCACAGCCTGAGG-3’; neo sense: 5’-GTGGGATTAGATAAATGCCTGCTC-3’. To determine the expression levels of TLR5 gene in knockout and wild type mice, Trizol reagent (Invitrogen, Carlsbad, CA) and RNeasy columns with DNase I treatment (Qiagen, Valenica, CA) were used to isolate total RNA from tissue samples (adipose tissue and colon). The cDNA was synthesized by VILO kit (Invitrogen, Carlsbad, CA) following the manufacturer’s instructions. StepOnePlus^TM^ 96-well machine (Applied Biosystems, Foster City, CA) was used for real-time quantitative PCR analysis (RT-PCR). The PCR products were detected using Sybr Green (Applied Biosystems, Foster City, CA) and normalized to 18S ribosomal RNA, using specific oligonucleotides with the following sequences: for mouse 18S, 5’-GGAGGATGAGGTGGAGCGAGT-3’(forward) and 5’-GCCTCTCCAGGTCCTCACGC -3’ (reverse); for mouse TLR5, 5’-AAGACTGCGATGAAGAGGAAGCCA -3’ (forward), 5’-TGTCCTTGAACACCAGCTTCTGGA-3’ (reverse). PCR products were confirmed by agarose gel.

### Determination of body composition, serum insulin, blood glucose, and free fatty acid concentrations

Body composition (lean mass & fat mass) was determined by quantitative magnetic resonance (QMR) in the Animal Physiology Core facility of the University of Alabama at Birmingham Diabetes Research Center. Serum insulin levels were measured using an ultra-sensitive mouse insulin ELISA kit (Crystal Chemicals, Inc. Downers Grove, IL) according to the manufacturer’s protocols. Blood glucose levels were determined by AlphaTrack glucose monitoring system (Abbott Animal Health, Chicago, IL). The free fatty acids were determined using enzymatic colorimetric assays (Wako, Richmond, VA) according to the manufacturer’s protocols.

### Glucose tolerance test and insulin tolerance test

Glucose tolerance tests (GTT) and insulin tolerance tests (ITT) were employed to evaluate the glucose tolerance and insulin sensitivity of KO and WT mice. Tests were done at indicated times in the life cycle, and before and after high fat diet feeding. To determine glucose tolerance, animals were fasted overnight and then given an intraperitoneal injection of glucose solution (100 g D-glucose/L; 2g/kg body weight), and glucose concentrations were measured in mouse tail blood at baseline (prior to injection), and at 30, 60, 90, 120 and 150 min post-injection using a AlphaTrack glucose monitoring system (Abbott Animal Health, Chicago, IL). To determine insulin tolerance as an assessment of insulin sensitivity, mice were fasted for 6 h in the morning of the test day and then administered an intraperitoneal injection of insulin solution (1–1.5 U insulin/kg body weight, HUMALOG® (insulin lispro injection, USP [rDNA origin]), Eli Lilly, Indianapolis, IN). Glucose levels were similarly monitored as described above in the glucose tolerance testing.

### Sequencing and analysis of gut microbiome

Littermate mice were separately housed based on genotypes. To assess the gut microbiome, genomic DNA was extracted from the cecal contents of WT or TLR5KO2 mice (n = 10 per group) using ZR Fecal DNA Kit (ZYMO research corp. Cat#: D6010) and stored in -80 freezer until used for sequencing. Pyrosequencing of the 16S RNA gene was performed on microbiota DNA samples using the bacterial tag-encoded GS FLX-Titanium amplicon with primers 28f (5’- GAGTTTGATCNTGGCTCAG-3’) and 519r (5’-GTNTTACNGCGGCKGCTG-3’). The sequence data are uploaded into the Sequence Read Archive (SRA) (http://www.ncbi.nlm.nih.gov/sra) and accessible through the accession number SRR3114140. Sequences were processed with the *QIIME* software package[9]. Briefly, barcodes and primers were depleted and sequences with an average quality score of less than 30 were removed from the dataset. Sequences shorter than 200 base pairs, containing ambiguous base-pair designation or greater than 8 homopolymers were also removed to maintain sequencing quality and aligned to the V1-V3 region of bacterial 16S RNA gene using the SILVA reference alignment as a template[10]. Chimeric sequences were removed using the UCHIME algorithm[11]. A distance matrix was created with a threshold of 0.15 and was used to cluster sequences into operational taxonomic units (OTU) using the Mothur software package algorithm for average neighbor grouping with a cutoff of 95% sequence similarity. Finally, OTUs were classified into consensus taxonomies using the SILVA database. Within community diversity (α-diversity) was calculated using QIIME. Alpha rarefaction curve was generated using Chao estimator of species richness[12] with ten sampling repetitions at each sampling depth. An even depth of 1,500 sequences per sample was used for calculation of richness and diversity indices. To compare microbial composition between samples, β-diversity was measured by calculating the weighted and unweighted Unifrac distances[13] based on proportion of OTUs and phylogenetic relation between those OTUs. Principal coordinate analysis (PCoA) was applied on resulting distance matrices using PRIMER v6 software[14]. Permutational multivariate analysis of variance (PERMANOVA)[15] was used to calculate P-values and test for significant differences of β-diversity among treatment groups. Label permutations were used in PERMANOVA to estimate the distribution of test statistics under the null hypothesis that within-group distances are not significantly different from between-group distances. Both weighted and unweighted Unifrac distances were used to compute the test statistic and P-values to determine the significance of the statistic[16].

### Metagenomic imputation of bacterial community

We used PICRUSt (Phylogenetic Investigation of Communities by Reconstruction of Unobserved States, http://picrust.github.com) [17], a bioinformatics tool that predicts gene family based on 16S gene surveys for bacterial community. For the analysis, OTUs were closed-reference picked against updated Greengenes database (reference) using QIIME v1.8 according to the online protocol [17, 18]. The resulting closed-reference OTU table was then input into the PICRUSt pipeline. The accuracy of metagenome predictions was judged by how closely related the microbes in a given sample are to microbes with sequenced genome representatives, as measured by Nearest Sequenced Taxon Index (NSTI), with lower values indicating a closer mean relationship[17, 18]. Analyzing PICRUSt predicted metagenomes was implemented in a graphical software package, STAMP (Statistical Analysis of Metagenomic Profiles, http://kiwi.cs.dal.ca/Software/STAMP [19]. Principal coordinate analysis (PCoA) was applied on distance matric of resulting functional abundances using PRIMER v6 software[14]. Permutational multivariate analysis of variance (PERMANOVA)[15] was used to calculate P-values and test for significant differences of functional diversity among treatment groups.

### Statistical methods

Data in bar figures are presented as means with S.E.M. Statistical significance was assessed by Student’s two-tailed t test or ANOVA, with p-values of <0.05 considered as significant. Taxonomically classified OTUs for each sample were converted into percentage data at the phylum and the genus levels. Relative abundances were tested for normality of distribution using PROC Univariate, and if normal, were analyzed using PROC Mixed in SAS (SAS Institute Inc, Cary, NC) with treatment as fixed and animal as random factors. Data that were not normally distributed were treated with PROC GLIMMIX through either Poisson or negative binomial distributions; with the Pearson chi-square / degrees of freedom ratio being applied to determine goodness of fit for each non-normal distribution method. Relative abundances were also subjected to partial least squares discriminant analysis (PLS-DA; SIMCA P+ 13.0 software package, Umetrics, Umea, Sweden), a supervised methodology that is a combination of principal component analysis (PCA) and regression, to analyze the differences in gut microbiome composition among treatments. The Y variables were used to describe the treatments, while X variables were used to represent the bacterial genera. The number of significant components was determined using R^2^ (indicator of goodness of fit) and Q^2^ values (indicator of predictive value of the model). Variable influence on projection value (VIP) was determined for each genus, and any with a VIP value below 0.3 was removed from the model. Score scatter plots and loading scatter plots were generated, and genera significantly associated with either treatment were determined by the PLS-regression coefficients and their plots.

## Results

### Metabolic features of TLR5KO2 mice

#### Body weight, food intake & body composition

**Fig 1:**
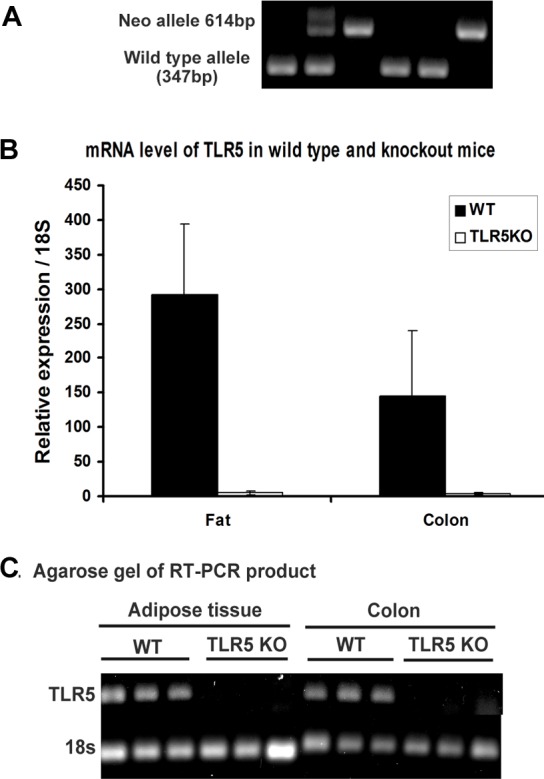
Generation of TLR5 knockout mice. A: genotyping results (WT-wild type, HT-heterozygous, KO-knockout); B: Real-time PCR quantified mRNA levels of TLR5 in wild-type (WT) and knockout mice (TLR5KO), adipose tissue and colon tissue were shown. C: Real-time PCR products were run on 1% agarose gel and confirmed knockout of TLR5.

**Fig 2:**
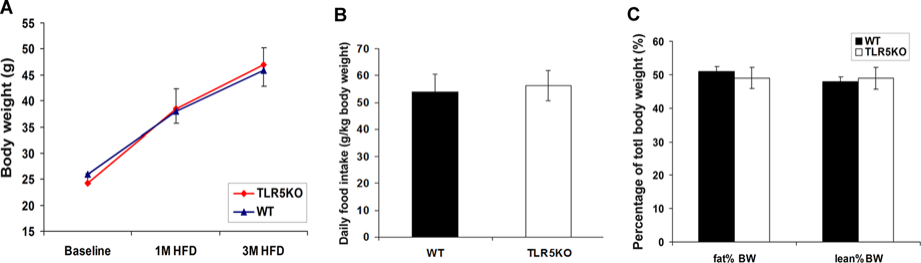
Body weight, food intake and body mass composition in wild type (WT) and TLR5 knockout (TLR5KO) mice fed a high fat diet. A: mice were fed with 12 weeks of high fat diet starting at 8 weeks of age (n = 20 per group). Body weight was monitored as indicated. B: Mice were individually caged and adapted for one week. Food intake (HFD) was measured manually every other day for a total period of 10 days. Daily food intake was calculated from data collected for a period of 10 days (n = 6 per group). C: Body fat mass and lean mass of HFD-fed mice was measured by quantitative magnetic resonance (QMR) (n = 8 per group).

In the previous study[7], TLR5KO1 mice developed higher body weights than those of their WT littermates beginning at 4 weeks of age, and continued to exhibit significantly higher weights over their life course. The current study has generated a second colony of TLR5KO2 and was aimed to explore the mechanistic links between gut microbiome and metabolic syndrome in TLR5 deficient mice (Fig 1). However, in the current study, body weights increased equally and were similar over time in wild type and TLR5KO2 groups when fed normal chow. Similarly, a high fat diet led to increments in body weight in both WT and TLR5KO2 mice without a significant difference between genotypes (Fig 2 and S1 Fig). Food intake was also similar between WT and TLR5KO2 mice (Fig 2). Body composition measured by QMR showed no significant differences in lean mass or fat mass between genotypes (Fig 2).

#### Fasting blood glucose, serum insulin & lipid profile

**Fig 3:**
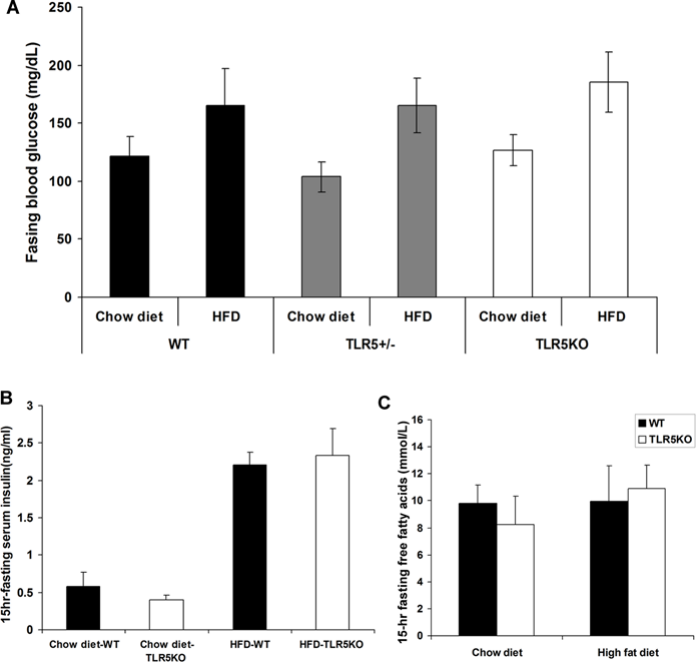
Fasting blood glucose, serum insulin, and lipid profile. A: 15hr fasting blood glucose was measured in wild-type (WT), heterozygous (TLR5+/-), and homozygous TLR5 knockout mice (TLR5KO) fed with either normal chow or a high fat diet. B: serum samples were collected in mice fed with normal chow diet or high fat diet, and insulin levels were measured using-sensitive mouse insulin ELISA kit. C: serum free fatty acids were measured in 20-week-old mice fed with either chow diet or high fat diet for 12 weeks.

In the previous study[7], TLR5KO1 mice showed statistically elevated blood glucose levels compared to WT littermates and basal insulin levels were also significantly increased. In the current study, 8 week old TLR5KO2 and WT mice had similar fasting glucose levels on normal chow (122.88±25.96 and 124.57±23.02 mg/dl respectively; p = NS). These mice were then placed on a high fat diet for additional 12 weeks and again TLR5KO2 and WT mice were found to have similar fasting glucose values (167.46±38.69 and 153.63±35.18 mg/dl; p = NS) (Fig 3 and S1 Fig), fasting serum insulin, and fasting free fatty acids levels (Fig 3). The results were consistently similar in non-segregated mice (S1 Fig).

#### Glucose-tolerance test & Insulin tolerance test

**Fig 4:**
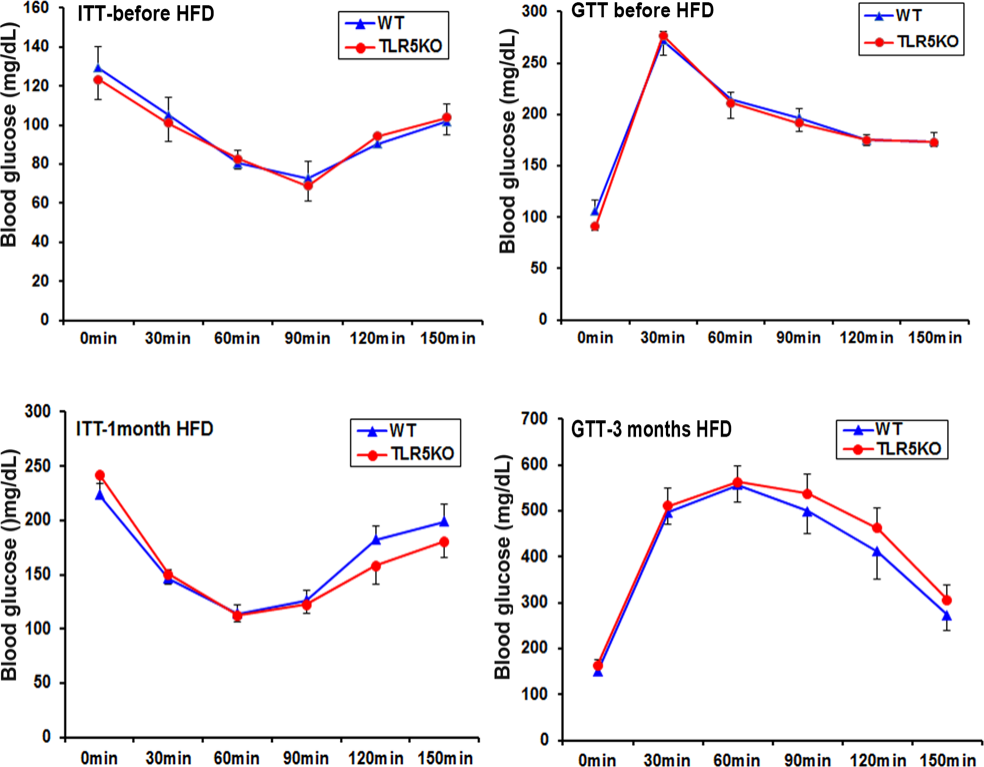
Glucose tolerance tests (GTT) and insulin tolerance tests (ITT) in wild type (WT) and TLR5 knockout (TLR5KO) mice. Insulin sensitivity was evaluated in 20-week-old mice before or after the high fat diet feeding for 12 weeks. To determine glucose tolerance, animals were first fasted overnight and then given an intraperitoneal injection of glucose solution (100 g D-glucose/L; 2g/kg body weight) and glucose concentrations were measured in mouse tail blood at baseline (prior to injection), and at 30, 60, 90, 120 and 150 min post-injection using a AlphaTrack glucose monitoring system (Abbott Animal Health, Illinois). To determine insulin tolerance, mice were fasted for 6 h in the morning of the test day and then administered an intraperitoneal injection of insulin solution (1–1.5 U insulin/kg body weight). Glucose levels were similarly monitored as described above in the glucose tolerance testing (n = 6–10).

On a normal chow diet, no difference was observed in the GTT and ITT results when comparing TLR5KO2 versus WT mice (Fig 4 and S1 Fig). To investigate effects of diet-induced obesity, we fed the mice a high-fat diet. As expected, both TLR5KO2 and WT mice exhibited increments in body weight and fat mass, as well as increased serum glucose and insulin levels; however, these values were not statistically different between the two genotypes. Similarly, glucose-tolerance and insulin tolerance test results were also similar in the TLR5KO2 and WT mice on a high fat diet (Fig 4 and S1 Fig). The results were consistently similar in non-segregated mice (S1 Fig).

### Analysis of gut microbial composition in TLR5KO2 and WT littermates

The lack of metabolic syndrome traits in TLR5KO2 mice, led us to the hypothesis that different gut microbial compositions could explain the lack of a metabolic syndrome phenotype in the current experiments in contrast to results previously reported in TLR5 deficient mice[7]. Based on alpha-diversity analysis, all measured indices between wild type and TLR5 deficient mice are not significantly different in both previous (TLR5KO1) and current (TLR5KO2) studies (*P>0*.*05*, data not shown). Under chow diet feeding, the microbiota in both wild type and TLR5KO2 featured a high percentage of Firmicutes (88%) and low percentage of Bacteroidetes (5–6%), without significant difference between WT and KO groups. This composition is dramatically different from the percentages in the previous TLR5KO1 study, in which Bacteroidetes predominate as 40.21% and 44.31% of the gut microbiota in wild-type mice and TLR5KO1 mice fed with chow diet, respectively (Table 1). The proportions of Proteobacteria and Tenericutes were significantly different between KO1 and KO2 study, the proportions of Actinobacteria and Deferribacteres are also noticeably different between the TLR5KO1 and TLR5KO2 mice in their respective studies (Table 1). Proteobacteria were decreased in both genotypes in the TLR5KO2 study as compared with WT in TLR5KO1 study. By contrast, all the other “minority phyla” including Tenericutes, Actinobacteria and Deferribacteres were increased in both genotypes in the TLR5KO2 study as compared with TLR5KO1 study (Table 1).

**Table 1:** Relative abundances of bacterial phyla in the fecal microbiota in the TLR5KO1 study and the TLR5KO2 study split by TLR5 knock-out mice and wild-type mice.

Phyla Present	Percentages Sequences in:^1^^,^^2^				SEM	*P*-value^3^
	TLR5-KO1		TLR5-KO2			
	TLR5-KO	WT	TLR5-KO	WT		
	-------------------------------above 1% of population----------------------------					
Bacteroidetes	44.31^a^	55.07^a^	5.15^b^	5.75^b^	3.09	<0.0001^G^
Firmicutes	52.93^b^	40.21^C^	88.13^a^	88.36^a^	2.81	<0.0001^G^
Other	1.27^ab,A^	0.19^b,B^	1.53^a,A^	1.74^a,A^	0.28	0.0051^G^
Proteobacteria	1.18^ab,B^	4.22^a,A^	1.00^b,B^	0.66^b,B^	0.55	0.0049^P^
Tenericutes	0.22^AB^	0.10^B^	3.03^A^	2.63^A^	0.46	0.025^P^
	------------------------between 0.1 and 1% of population---------------------					
Actinobacteria	0.03	0.11	0.71	0.53	0.2305	0.4433^P^
Deferribacteres	4.16E-07	4.16E-07	0.32	0.20	0.113869	0.9875^P^
TM7	0.06	0.10	0.13	0.13	0.05247	0.7586^G^

Note:TLR5KO2 mice refer to the TLR5 knockout mice in the current study and TLR5KO1 mice to a different colony of TLR5 knockout mice previously studied by Qin et al[7].

^1^ Statistically different (*P*<0.05) denoted by ^a,b,c^

^2^ Statistically different (*P*<0.10) denoted by ^A,B,C^

^3^ Method of analysis denoted by ^G^ (Gaussian), and ^P^ (Poisson).

**Fig 5:**
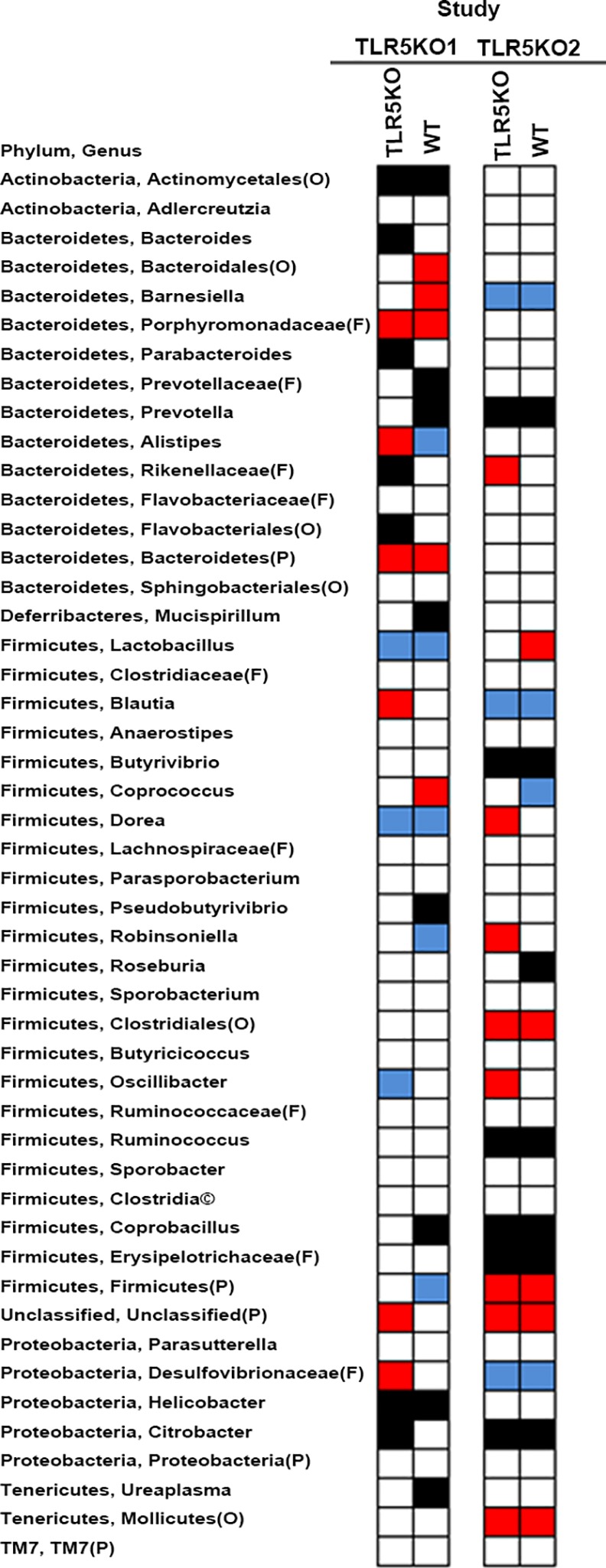
Impact of TLR5 knockout on specific taxa in the fecal microbiota of mice and differences in comparing two colonies (TLR5KO1 and TLR5KO2) bred in different laboratories. Heat map showing statistically significant changes (p<0.05) on different fecal taxa. Red shows a significant increase, blue signifies a significant decrease, and black shows not found. Bacterial taxa were obtained from QIIME. When an OTU could not be classified to the genus level, the closest level of classification was given, preceded by F (family), O (order), C (class), or P (phylum). Note: TLR5KO2 mice refer to the TLR5 knockout mice in the current study and TLR5KO1 mice to a different colony of TLR5 knockout mice previously studied by Vijay et al[7].

There were also significant differences regarding the diversity of microbiota between the two studies (Fig 5). Regarding Bacteroidetes in the TLR5KO1 study, there were a number of taxa that were non-detectable although the identity of the non-detected taxa differed between WT and KO mice, indicative of a genotype effect on bacterial diversity. Specifically, several members of Bacteroidetes including *Bacteroides*, Parabacteroides, Rikenellaceae(F), and Flavobacteriales(O) were non-detectable in TLR5KO1 mice; by contrast, Prevotellaceae(F) and *Prevotella* were non-detectable in WT mice. Additionally, Bacteroidales (O) and *Bamesiela* were increased in WT, while *Alistipes* was increased in TLR5KO1 but decreased in WT (Fig 5). However, these results stand in contrast with the current TLR5KO2 study where differences in Bacteroidetes between WT and TLR5KO were not consistently observed against the background of a dramatic reduction in total Bacteroidetes (Table 1 and Fig 5). Although the percentage of overall Firmicutes phylum was augmented in both WT and KO mice, the diversity of genera within Firmicutes phylum was diminished in TLR5KO2 study. For example, *Butyrivibrio*, *Ruminococcus*, *Coprobacillus*, and Erysipelotrichaceae(F) were not detectable in both WT and KO mice, additionally, *Roseburia* was absent in WT mice (Fig 5).

**Fig 6:**
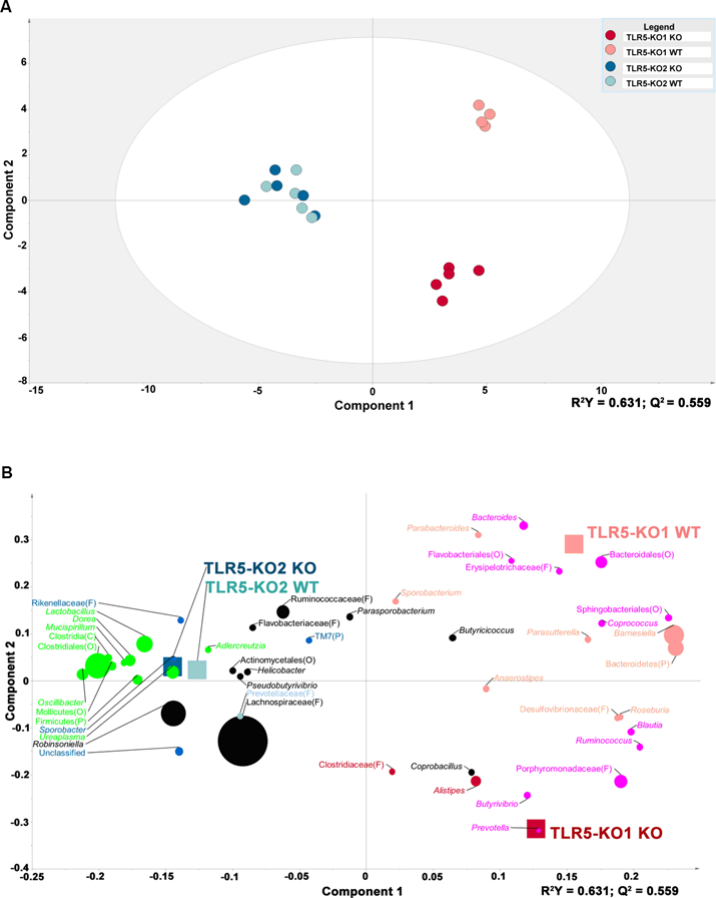
Partial least squares discriminant analysis (PLS-DA) graphs of the differences in gut microbial composition between the TLR5KO1 study and TLR5KO2 study. Pyrosequencing data was subjected to PLS-DA. A: Score scatter plot representing individual animals from each study, grouped based on the composition of fecal microbiota. The R2 and Q2 of the model were 0.63 and 0.56, respectively. B: Bacterial taxa plotted using weighted PLS component 1 and 2. Genera in the plot closer to either treatment are more strongly associated to it. Genera found to significantly contribute to the model prediction are shown in light red (TLR5KO1 wild-type), dark red (TLR5KO1 knock-out), light blue (TLR5KO2 wild-type), and blue (TLR5KO2 knock-out). Genera that significantly contributed to both groups in TLR5KO1 were shown in pink, and genera that significantly contributed to both groups in TLR5KO2 study were shown in green. When a sequence could not be classified to the genus level, the closest level of classification was given, preceded by F (family), O (order), C (class), or P (phylum). Note: TLR5KO2 mice refer to the TLR5 knockout mice in the current study and TLR5KO1 mice to a different colony of TLR5 knockout mice previously studied by Qin et al[7].

**Fig 7:**
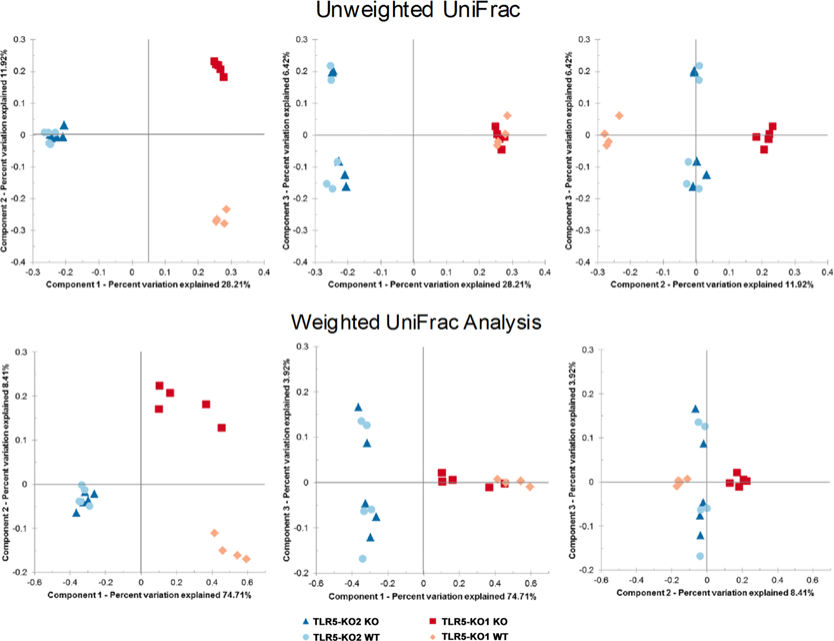
PCOA graphs of the differences in gut microbial composition between the TLR5KO1 study and TLR5KO2 study. Pyrosequencing data was analysed with QIIME and subjected to both unweighted and weighted Unifrac analysis. Top panels: Unweighted Unifrac analysis, component 1 explains 28.21% of the variation, component 2 explains 11.92% of the variation, and component 3 explains 6.42% of the variation. Bottom panels: Weighted Unifrac analysis, component 1 explains 74.71%% of the variation, component 2 explains 8.41% of the variation, and component 3 explains 3.92% of the variation.

To further identify the differences in the gut microbial compositions between the TLR5KO1 study and the TLR5KO2 study, individual animals were grouped based on the composition of fecal microbiota via partial least squares discriminant analysis (PLS-DA). The WT and TLR5KO1 mice in the TLR5KO1 study were distinctly grouped based on the composition of fecal microbiota, which confirmed the different microbial compositions in these mice. However, this distinct grouping was not present in TLR5KO2 mice; instead, WT and TLR5KO2 mice overlapped demonstrating no significant differences in the fecal microbial compositions (Fig 6). Bacterial taxa plotting further revealed that the dramatic genera differences between the WT and TLR5KO mice found in the TLR5KO1 study were not observed between the WT and TLR5KO mice in TLR5KO2 study (Fig 6 and Table 2). For instance, in the TLR5KO1 study there were 8 genera significantly associated with WT mice, however, none of which was associated with WT mice in the TLR5KO2 study (Fig 6 and Table 2). The genera that associate with both genotypes in TLR5KO2 study (green plots) are also totally different from the genera found to be associated with both genotypes in the TLR5KO1 study (pink plots) (Fig 6 and Table 2). Principal coordinate analysis (PCoA) was used to compare the beta-diversity of microbiota in the TLR5KO2 study with the TLR5KO1 study. As shown in Fig 7 the first two principal coordinates of analysis (component 1 and 2) separated mice colonies in both the TLR5KO1 study and in the TLR5KO2 study. Based on PERMANOVA analysis, there was a clear separation between KO and WT mice in the TLR5KO1 study (*P = 0*.*01* for unweighted and *P = 0*.*012* for weighted Unifrac), but no separation between KO and WT mice in the TLR5KO2 study, supporting similar diversity of gut microbiome between KO and WT in the current study (*P = 0*.*97* for unweighted and *P = 0*.*99* for weighted Unifrac).

**Table 2:** List of genera found predominantly contribute to WT, KO, or both genotypes in TLR5KO1 and TLR5KO2 study (data is also presented in Fig 6).

	TLR5KO1 study	TLR5KO2 study
**Genera contribute to WT**	Bacteroidetes,Bacteroidetes	Bacteroidetes,Prevotellaceae(F)
	Bacteroidetes,Barnesiella	
	Bacteroidetes,Parabacteroides	
	Firmicutes,Anaerostipes	
	Firmicutes,Sporobacterium	
	Firmicutes,Roseburia	
	Proteobacteria,Desulfovibrionaceae(F)	
	Proteobacteria,Parasutterella	
**Genera contribute to KO**	Bacteroidetes,Alistipes	Bacteroidetes,Rikenellaceae(F)
	Firmicutes,Clostridiaceae(F)	Firmicutes,Sporobacter
		TM7(P)
		Unclassified
**Genera contribute to both WT & KO**	Bacteroidetes,Bacteroides	Firmicutes,Clostridiales(O)
	Bacteroidetes,Bacteroidales(O)	Firmicutes,Clostridia(C)
	Bacteroidetes,Flavobacteriales(O)	Firmicutes,Dorea
	Bacteroidetes,Porphyromonadaceae(F)	Firmicutes,Firmicutes(P)
	Bacteroidetes,Prevotella	Firmicutes,Lactobacillus
	Bacteroidetes,Sphingobacteriales(O)	Firmicutes,Oscillibacter
	Firmicutes,Blautia	Actinobacteria,Adlercreutzia
	Firmicutes,Butyrivibrio	Deferribacteres,Mucispirillum
	Firmicutes,Coprococcus	Tenericutes,Mollicutes(O)
	Firmicutes,Erysipelotrichaceae(F)	Tenericutes,Ureaplasma
	Firmicutes,Ruminococcus	

### Predicted metabolic functions in both genotypes of TLR5KO1 and TLR5KO2 study

**Fig 8:**
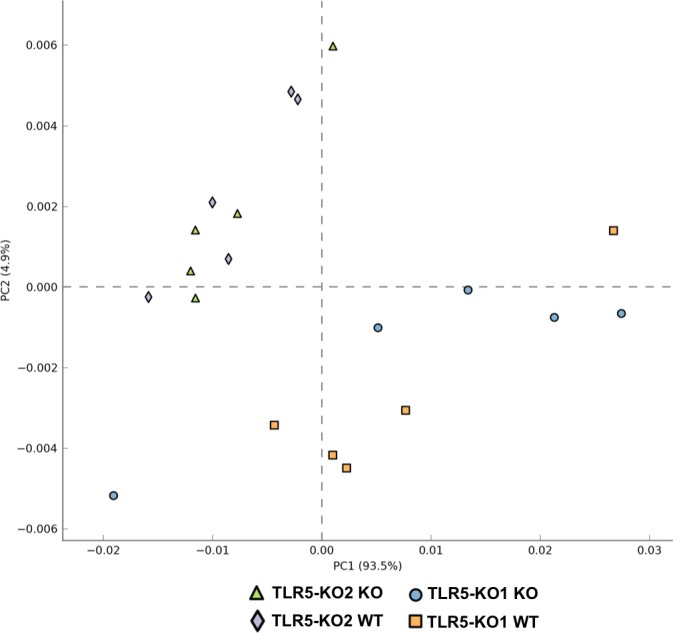
Principle component analysis (PCA) graph of the differences in the gut microbial metagenomic compositions between the TLR5KO1 study and TLR5KO2 study. Metagenomic functions were predicted using 16S bacterial profile data implemented in PICRUSt pipeline and predicted KEGG functional genes (at level 3) were analyzed with Kruskal-Wallis H-test to generate PCA plot. Component 1 explains 93.5% of the variation, and component 2 explains 4.9% of the variation.

**Fig 9:**
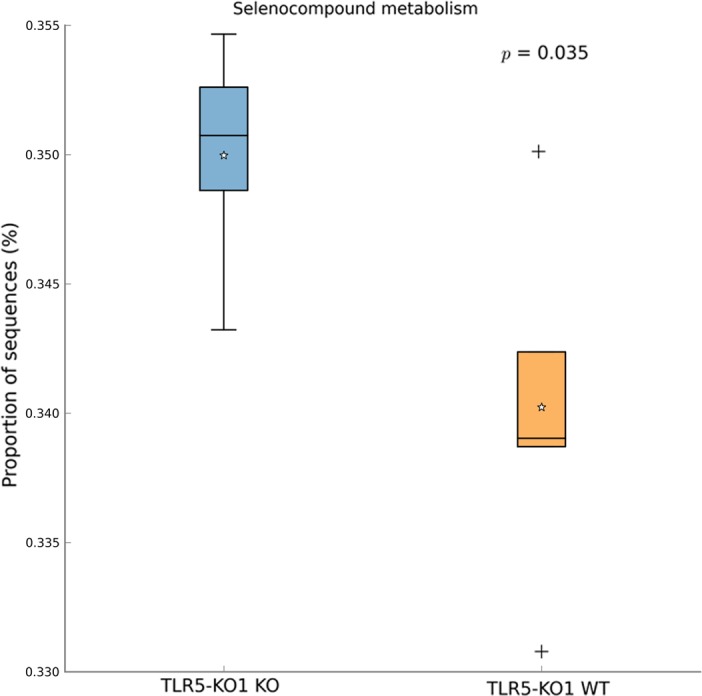
A box plot showing significant differences in selenocompound metabolism of the gut microbiome between WT and KO mice in the TLR5KO1 study. Predicted metagenomic functions of bacterial community were compared between WT and KO mice of TLR5-KO1 study (see reference number 8). Welch’s t-test was used to identify the statistically significant differences between genotypes (P<0.05).

In addition to microbiota profiles, we further conducted metagenomic functional analysis and highlighted certain pathways associated with metabolic syndrome. Weighted Nearest Sequenced Taxon Index values were used to assure the accuracy of metagenome predictions. In this analysis, the weighted NSTI value was 0.19±0.03. The principle component analysis (using Kruskal-Wallis H-test) generated from predicted metagenomic functions showed significant difference between TLR5KO1 and TLR5KO2 study (*P<0*.*05)*, similar to 16S profiles (Fig 8). Using PICRUSt as a predictive empirical tool, we found that there were no significant functional metagenomic differences between WT and KO mice in the TLR5KO2 study (*P = 0*.*34)*. However, a single level 3 KEGG Orthology group, selenocompound metabolism, was significantly difference between WT and KO mice in TLR5KO1 study (Welch’s t-test, P<0.05) (Fig 9). When comparing the corresponding genotype between both studies, we found that multiple predicted functions of the complete communities were different between WT mice of TLR5KO1 and WT from the TLR5KO2 study (113 KEGG orthology groups) (S2 Fig), as well as in KO mice of both studies (92 KEGG orthology groups) (S3 Fig).

### Classification of bacterial taxa those were associated with metabolic syndrome

The metabolic assessment revealed no phenotypic difference between WT and KO mice in the TLRKO2 study; however, differences on metabolic features were significant between WT and KO mice in the TLR5KO1 mice. By comparing the microbiota information in the TLR5KO1 study and TLR5KO2 study, we next endeavored to identify specific microbiota that could be the true positive candidates associated with the metabolic syndrome in the TLR5KO1 mice. We have conducted additional analyses (Table 1 and Fig 5) based on the following inclusion/exclusion criteria: In the TLR5KO1 study: 1) Bacterial taxa with relative increase or decrease in KO mice as compared with WT mice could be the candidates associated with the metabolic syndrome; 2) Microbiota that changed equally in both genotypes are not likely to be responsible for the metabolic differences between KO and WT mice. In order to rule out non-specific changes of microbiota in TLR5KO1 mice, candidate taxa identified from the above analysis are further filtered using data from the TLR5KO2 study as follows: 1) Taxa that relatively increased or decreased in KO of TLR5KO1 study but showed no or even opposite changes in TLR5KO2 study remain as strong candidates related with metabolic syndrome; 2) Taxa that are anti- or pro- metabolic disorders could be increased or decreased in both genotypes in TLR5KO2 study, since this could explain the absence of phenotypic differences between genotypes in the TLR5KO2 study. Based on these analyses using data from the current and previous [8] studies, we identified certain microbiota as potential true positives and strong candidates associated with the metabolic syndrome, shown in Table 3.

**Table 3:** Bacterial changes specifically associated with metabolic syndrome based on a comparison of gut microbiota in WT mice and two colonies of TLR5KO mice, only one of which has the metabolic syndrome.

Relatively higher abundance in the metabolic syndrome	Relatively lower abundance in the metabolic syndrome
Bacteroidetes, Alistipes	Bacteroidetes, Bacteroides
Bacteroidetes, Prevotella	Bacteroidetes, Bacteroidales(O)
Bacteroidetes, Prevotellaceae(F)	Bacteroidetes, Barnesiella*
Firmicutes, Blautia	Bacteroidetes, Parabacteroides
Firmicutes, Butyrivibrio	Bacteroidetes, Rikenellaceae(F)
Firmicutes, Coprobacillus	Bacteroidetes, Flavobacteriales(O)
Firmicutes, Erysipelotrichaceae(F)	Firmicutes, Clostridiales(O)
Firmicutes, Pseudobutyrivibrio	Firmicutes, Coprococcus
Firmicutes, RobinsonielLa	Firmicutes, Oscillibacter
Firmicutes, Ruminococcus	Proteobacteria, Citrobacter*
Deferribacteres, Mucispirillum	Tenericutes, Mollicutes(O)
Proteobacteria, Desulfovibrionaceae(F)	
Tenericutes, Ureaplasma	

* Note: these genera were decreased in KO1 mice which had metabolic syndrome, however, in the current study, reduction of these genera did not cause metabolic syndrome, which might be a result of compensation from other genera or lacking of other microbiome profiles that are required for development of metabolic syndrome.

## Discussion

The gut microbiome consists of approximately 500–1,000 OTUs which can be generally grouped into seven bacterial phyla. The most abundant phyla found in the human intestinal tract are Firmicutes and Bacteriodes, and other minor phyla colonized in human gut include Actinobacteria, Cyanobacteria, Fusobacteria, Proteobacteria, and Verrucomicrobia[20, 21]. Both genetic and environmental factors act to determine the identity and distribution of the phyla and species of gut bacteria. Colonization begins during the passage of the fetus through the mother’s vagina or skin contact, after which, breast feeding, as well as subsequent environmental exposures modify gut microbiota composition[22]. The role of environmental factors in modulating gut microbiota composition has been strongly indicated by the observation that co-habiting parents share significantly more similar microbiota composition compared with individuals from other families[23]. Important environmental factors include diet, lifestyle/exercise, individual hygiene, and use of medications and antibiotics, all of which collectively participate in colonization, adaption, and stabilization of gut microbiota in the host[24]. Regarding genetic factors, Toll-Like Receptors (TLRs) are major mediators of the interactions between gut microbiota and host innate immune system[25]. In particular, it was reported in mice that gene deletion of TLR5 (TLR5KO1), which is expressed in intestinal mucosa and recognizes bacterial flagellin, leads to an alteration in gut microbiota that is coupled to the development of Metabolic Syndrome, and which can confer this metabolic phenotype upon transference of TLR5KO1 gut microbiota to WT mice [8]. Our paper addresses the relative importance of environment and genetics to both the microbiome and metabolism in the TLRKO mouse model.

We have now studied another line of TLR5KO mice (TLR5KO2), and we have found that microbiota compositions remained similar between TLR5KO2 and WT, as well as their in silico inferred metagenomics functions. Furthermore, there were no significant metabolic differences between TLR5KO2 and WT mice whether being fed normal chow or a high fat diet. This is in direct contradistinction from the earlier report in another TLR knockout mouse line (TLR5KO1), where an increase in the Firmicutes/Bacteroides (F/B) ratio and an insulin resistance phenotype consistent with the metabolic syndrome was observed only in the KO mice[8]. In our study, it is important to consider that there was a pronounced increase in Firmicutes in both TLR5KO2 (88.13%) and WT littermates (88.36%), as well as the Firmicutes/Bacteroides ratio, which could lead to development of insulin resistance in both TLR5KO2 and WT mice without significant difference between genotypes. This consideration is based on the observation that a high Firmicutes/Bacteroides ratio was similarly observed in Ob/Ob mice, an obese insulin-resistant model exhibiting the metabolic syndrome[26, 27]. Even so, future studies are needed to establish a conclusive role regarding the contribution of total Firmicutes/Bacteroides ratio, and the relative abundance of bacterial taxa within each phylum, in the development of disease. Even so, the current data indicate that environment can predominate over genotype in determining gut microbiota composition and microbiota-dependent effects on systemic metabolism.

In previous studies, Ubeda et al. demonstrated that intestinal microbiota remained stable in the absence of TLRs (TLR5 as well as TLR2, TLR4, and TLR9) when compared with the WT littermates, indicating that colonization of microbiota could have mainly resulted from specific maternal and environmental transmissions rather than defective TLR signaling[28]. In addition, Letran et al. found that TLR5KO mice did not exhibit any metabolic differences with WT although the KO mice did display T cell responses to a flagellated pathogen. While the combination of metabolic phenotype and gut microbiota were not both studied in each of these earlier reports, in aggregate the data indicate that TLR5KO mice may not be associated with any alterations in gut microbiota or metabolism.

Our data do not contradict the ideas that gut microbiota are necessary for human health and that changes in gut microbiota can adversely affect systemic metabolism. In addition to a roles in the metabolism of otherwise indigestible polysaccharides and synthesis of essential vitamins, the presence of a healthy gut microbiota is required for the normal development and differentiation of the host's intestinal epithelium and protection against invasion of the host by opportunistic pathogens[29]. On the other hand, many [27, 30, 31] but not all [32] studies have found that obesity is associated with increased Firmicutes combined with reductions in Bacteroides. Though the specific profile of obesogenic gut microbiota has not been identified, obese individuals also tend to have decreased microbiota diversity and altered microbiome encoding metabolic pathways such as carbohydrate metabolism and generation of short-chain fatty acids from the diet[33]. Alterations of gut microbial composition have also been associated with Type 2 diabetes[34]. While the promotion of healthy gut microbiota profile could be a novel and promising strategy to maintain health and prevent or treat disease, the current data suggests that it is the environment rather than the host genome that mainly determines gut microbiota composition.

In further analyses, we compared the microbiota data from the previous study in TLR5KO1 mice with our current data, to identify specific examples of differential expression between the mouse lines that did (TLR5KO1) and did not (TLR5KO2) exhibit the metabolic syndrome. Thus, we endeavored to identify specific microbial taxa that could be responsible for the metabolic phenotype (Table 3). Several salient points emerged, including: 1) TLR5KO2 mice had increased Firmicutes/Bacteroidetes (F/B) ratio compared with WT, however, the F/B ratio is strikingly elevated in both WT and knockout genotypes in TLR5KO2 study, and is even much higher than that of KO mice in the TLR5KO1 study. Together, the data indicate that the high F/B ratio originated from parent-generation or living environment rather than TLR5 deficiency per se. 2) Proteobacteria and Barnesiella were decreased in TLR5KO1 mice, indicating they potentially contribute to obesity and metabolic syndrome. The fact that reductions of Proteobacteria and Barnesiellain mice of both genotypes did not induce metabolic syndrome in the current study could result from compensation from other genera or lacking of other microbiome profiles that are required for development of metabolic syndrome. 3) In the current study, animals with high total Firmicutes and low total Bacteroidetes did not show obvious metabolic dysfunction, one possibility is that high Firmicutes/Bacteroidetes ratio at phylum level does not necessarily cause metabolic syndrome as reported in previous studies[2, 30, 35]. Additionally, instead of looking at the changes of total phyla, it is more important to monitor changes of specific genera within Firmicutes and Bacteroidetes phyla. In this scenario, only certain taxa may be specifically associated with disease, and the relative abundance of each genera within one phylum could be more important than changes at phylum level as listed in Table 3.

The potential associations between some of the identified taxa and metabolic regulation have been consistently supported or suggested by published studies. For instance, in a study on correlation between gut microbiota composition and fecal metabolic profiles in mice, authors found Barnesiella, Prevotella, and Alistipes were significantly decreased after antibiotic treatment; in addition, Alistipes was also positively correlated with fecal nutrient levels such as amino acids, organic acids and sugars. Interestingly, abundance of Prevotella, Alistipes, and Barnesiella are closely associated with the hosts’ capability of producing monosaccharide and short-chain fatty acids[36]. Very recently, a specific new species within the genus Alistipes was isolated from the fecal flora of a 26-year-old woman with morbid obesity[37]. Results suggest that lower abundance of Bacteroides might be associated with higher risk of metabolic syndrome, even if this decrement is unrelated to TLR5 deficiency. Consistent with this formulation, dietary porphyran (a water soluble fiber) improved glycemic control and significantly increased cecum Bacteroides in KK-Ay diabetic mice[38]. Gauffin et al. specifically reported that oral administration of Bacteroides uniformis CECT 7771 reduced body weight gain, liver steatosis, and liver cholesterol and triglyceride levels in HFD-fed mice[39]. Most recently, Roager et al. found that populations with central obesity and components of metabolic syndrome could be clustered into distinct groups simply by their relative abundance of Prevotella spp. divided by Bacteroides spp. (P/B ratio), and individuals with a high P/B ratio group had higher total plasma cholesterol[40]. Within the Firmicutes phylum, Blautia and Coprobacillus are two genera indicated by the current study to be potentially operative in the metabolic syndrome, and, in a study using computational method to analyze microbial community dynamics in rodents, other authors identified Blautia and Coprobacillus together with Akkermansia as the major representative genera that related with infection and maintenance of intestinal stability[41]. Based on our analysis, the relative abundance of specific minority genera which usually contribute a very small percentage to the total gut microbiome community have been implicated potential roles in the metabolic syndrome. These include relative increments in Mucispirillum, Proteobacteria-Desulfovibrionaceae (F), and Ureaplasma, and a relative decrease in Proteobacteria-Citrobacter. A previous rodent study has shown that increase of Mucispirillum was associated with high-fat feeding[42]. Change of abundance of Desulfovibrionacea (F) was also identified as a family responsible for the development of metabolic syndrome in animal models[43]. In a study done by Carvalho et al, imbalance of Proteobacteria has also been discussed as a stimulator of gut inflammation in TLR5-deficient mice[44]. Citrobacter was indicated as a major pathogen for necrotizing enterocolitis (NEC); however, its causative role for NEC has been controversial due to the complicated strain-specific physiology of citrobacter subpopulations[45].

In addition to microbiota profiles, predicted metagenomic functions also have the potential to implicate functional pathways in the pathogenesis of the metabolic syndrome. Based on multiple comparisons, we observed no statistically significant functional metagenomic differences between WT and TLR5KO2 mice in the current study, coupled with the absence of any metabolic differences between the WT and knockout mice. Products of microbial metabolism have been proposed as signaling molecules that can influence the host’s metabolism. Microbial metabolites directly affect gastrointestinal function but may also affect the liver, brain, as well as adipose and muscle tissues, which consequently may affect the level of obesity and metabolic syndrome[46]. In data from the previous TLR5KO1 study[7], we found that selenocompound metabolism was the only pathway differentially affected when comparing WT and TRL5KO1 mice. The toxicity of selenocompounds, especially selenite, has been observed in genetically obese rats (fa/fa) [47]. However, the underlying mechanisms by which microbial selenocompounds could influence metabolic syndrome traits have not yet been identified and deserve for further investigations.

The current study provided evidence that environment can trump genotype in modulating gut microbiota in a manner that affects systemic metabolism. While TLR5 deficiency per se had no impact, environmental explanations for the high Firmicutes/Bacteroidetes ratio in both TLR5-KO and WT mice could include transmission from the environment where the mice originated or from the environment where the animals were maintained for study that was routinely used for the study of diet-induced obesity or genetically obese animals. We did isolated KO mice from WT mice at the time of weaning to prevent cross-contamination between the gut microbiota of KO and WT mice and maintained the mice in isolated cages; however, this would not have precluded cross-contamination that might have occurred prior to weaning. Based on the current findings, researchers should be cautious on drawing conclusions from future studies, as the environment-induced alterations of gut microbiota could be a significant cofounding factor that causes falsely expanded or submerged inter-group differences.

## Supporting Information

S1 Fig Metabolic features of WT and KO mice under non-segregated housing condition. A: Body weight of mice fed on either chow diet or high fat diet for 12 weeks starting at 8 weeks of age. B: 15hr fasting blood glucose was measured in mice fed on either chow diet or high fat diet for 12 weeks starting at 8 weeks of age. C: Insulin sensitivity was evaluated by insulin tolerance test (ITT) in 20-week-old mice before or after the high fat diet feeding for 12 weeks. (n = 10–20)
(TIF)

S2 Fig Multiple predicted functions of the complete communities in WT mice in TLR5 KO1 and TLR5KO2 study. (TIF)

S3 Fig Multiple predicted functions of the complete communities in KO mice in TLR5 KO1 and TLR5KO2 study. (TIF)
