# Supplementary figures and images for: Deletion of the Toll-Like Receptor 5 Gene Per Se Does Not Determine the Gut Microbiome Profile That Induces Metabolic Syndrome: Environment Trumps Genotype
Source: PLoS One. 2016 Mar 7;11(3):e0150943. doi: 10.1371/journal.pone.0150943 (PMC4780789; doi:10.1371/journal.pone.0150943)

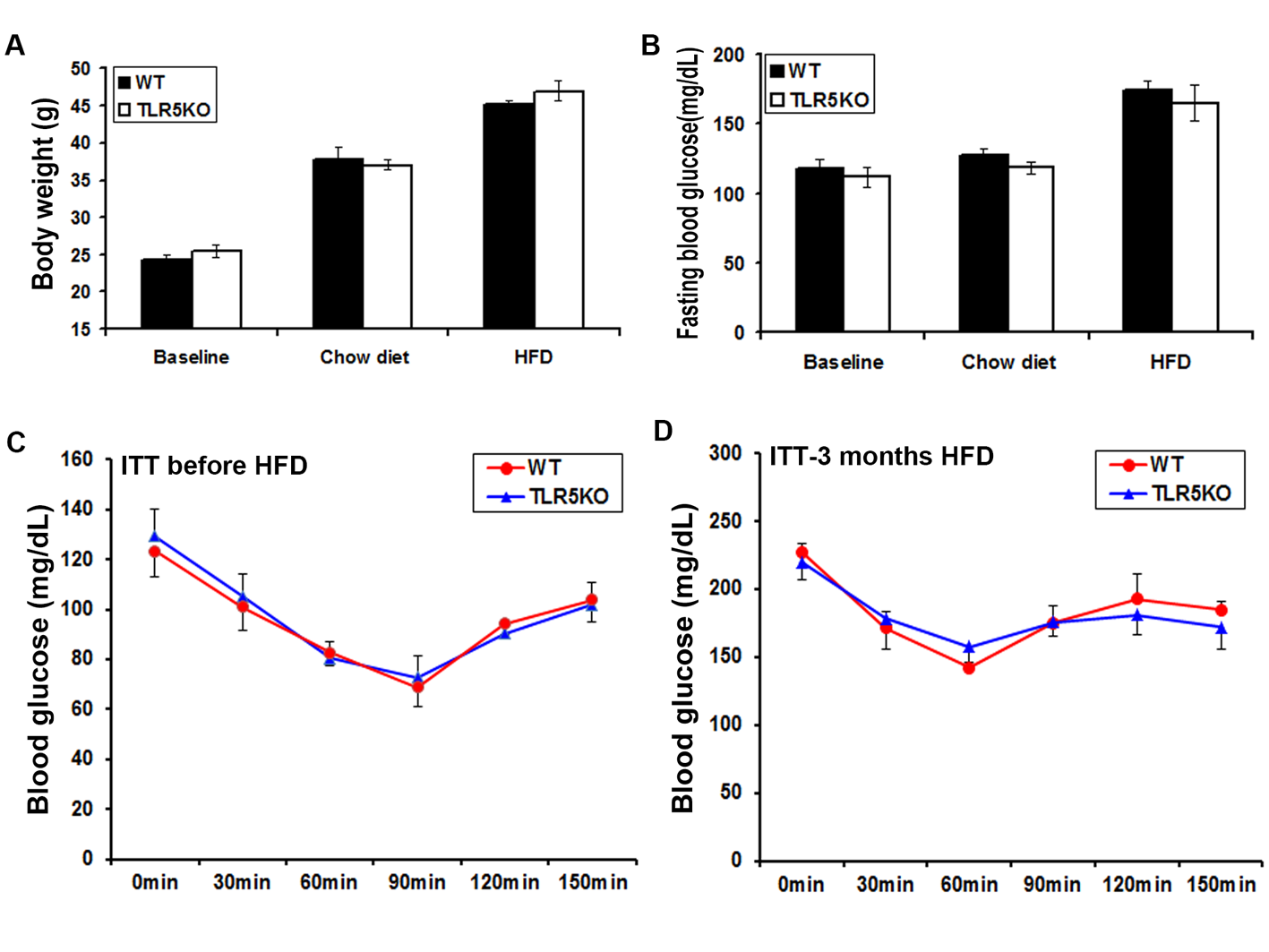

Supplement: S1 Fig — A: Body weight of mice fed on either chow diet or high fat diet for 12 weeks starting at 8 weeks of age. B: 15hr fasting blood glucose was measured in mice fed on either chow diet or high fat diet for 12 weeks starting at 8 weeks of age. C: Insulin sensitivity was evaluated by insulin tolerance test (ITT) in 20-week-old mice before or after the high fat diet feeding for 12 weeks. (n = 10–20) (TIF) [file pone.0150943.s001.tif]
